# Atheroregressive Potential of the Treatment with a Chimeric Monoclonal Antibody against Sulfated Glycosaminoglycans on Pre-existing Lesions in Apolipoprotein E-Deficient Mice

**DOI:** 10.3389/fphar.2017.00782

**Published:** 2017-11-01

**Authors:** Victor Brito, Katia Mellal, Karina F. Zoccal, Yosdel Soto, Liliane Ménard, Roger Sarduy, Lucia H. Faccioli, Huy Ong, Ana M. Vázquez, Sylvie Marleau

**Affiliations:** ^1^Faculté de Pharmacie, Université de Montréal, Montréal, QC, Canada; ^2^Division of Immunobiology, Center of Molecular Immunology, Havana, Cuba; ^3^Department of Clinical Analysis, Toxicology and Bromatology, Faculdade de Ciências Farmacêuticas de Ribeirão Preto, Universidade de São Paulo, Ribeirão Preto, Brazil

**Keywords:** proteoglycans, glycosaminoglycans, atherosclerosis, active immunization, regression, interleukin-6, interleukin-10, vascular cell adhesion molecule-1

## Abstract

The retention of lipoprotein particles in the intima, in particular to glycosaminoglycan side chains of proteoglycans, is a critical step in atherosclerosis initiation. Administration of chP3R99, a chimeric mouse/human monoclonal antibody inducing an anti-idiotypic network response against glycosaminoglycans was previously shown to prevent atherosclerotic lesion progression, yet its effect in the late-stage progression of lesions remains unknown. This study investigated the effect of chP3R99 at a late stage of disease development in apolipoprotein E-deficient mice and the vascular mechanisms involved. Male apolipoprotein E-deficient mice were fed a high-fat high-cholesterol diet from 4 to 19 weeks old, at which time mice were fed normal chow and 5 doses of chP3R99 (50 μg) or isotype-matched IgG (hR3) were administered subcutaneously weekly for the first 3 administrations, then at weeks 24 and 26 before sacrifice (week 28). Lesions progression was reduced by 88% in treated mice with no change in total plasma cholesterol levels, yet with increased sera reactivity to chP3R99 idiotype and heparin, suggesting the induction of an anti-idiotype antibody cascade against glycosaminoglycans, which was likely related with the atheroprotective effect. chP3R99 treatment initiated regression in a significant number of mice. Circulating levels of interleukin-6 were reduced along with a striking diminution of inflammatory cell accumulation in the vessel wall, and of VCAM-1 labeling *in vivo*. The ratio of IL-10/iNOS gene expression in aortas increased in chP3R99-treated mice. In conclusion, our results show that treatment with chP3R99 reduces vascular inflammatory burden and halts lesion progression with potential for regression in the late phase of the disease in atherosclerotic mice, and support the therapeutic intervention against glycosaminoglycans as a novel strategy to reverse atherosclerosis.

## Introduction

The subendothelial retention of basic apolipoprotein B (apoB)-containing lipoproteins through an electrostatic interaction with negatively charged glycosaminoglycan (GAG) side chains of proteoglycans is considered a key initiating step of atherogenesis (Williams and Tabas, 1995; Boren et al., 1998; Skalen et al., 2002; Tabas et al., 2007). In agreement, we previously reported the antiatherogenic properties of the chimeric murine/human monoclonal antibody (mAb) chP3R99, which binds sulfated GAGs, thus inhibiting low density lipoprotein (LDL)-chondroitin sulfate (CS) association and LDL oxidation (Brito et al., 2012; Soto et al., 2012, 2014). This mAb is highly immunogenic in mice and rabbits being its idiotype immunodominant despite the fact that 70% of chimeric molecules are xenogenic respect to animal models (Brito et al., 2012; Soto et al., 2012). Previous data have also shown that subcutaneous (*s.c*) administration of chP3R99 mAb (Ab1) in animal models generates an anti-idiotype antibody cascade in which a fraction of the induced anti-idiotype response (Ab2) behaves as a functional mimicry of GAGs epitopes, resulting in the induction of anti-anti-idiotype antibodies (Ab3) with the same specificity as Ab1, namely, recognition of sulfated GAGs (Brito et al., 2012; Soto et al., 2012). In concordance with this vaccine effect, the administration of chP3R99 to New Zealand White rabbits and hypercholesterolemic apoE^-/-^ mice, using a low-dose schedule applied simultaneously with lesion onset, reduced atherosclerotic lesions development. In both animal models, the antiatherosclerotic effect was likely associated to the induction of Ab3 antibodies that, as chP3R99, recognized proatherogenic GAGs and block lipoprotein retention (Brito et al., 2012; Soto et al., 2012). Further studies in apoE^-/-^ mice showed that delaying the low-dose *s.c* application of chP3R99 after 6 weeks of high-fat high-cholesterol (HFHC) feeding, halted the progression of aortic lesions and attenuated vascular inflammation and oxidative stress, effects also related to the generation of an anti-CS response in treated mice (Delgado-Roche et al., 2015).

Notwithstanding their role in early atherosclerotic lesion development, proteoglycans also play an important role in later stages of atherosclerosis where more complex mechanisms of lipoprotein retention occur such as changes in the expression pattern of proteoglycans to more retentive forms (Maor et al., 2000; Wight and Merrilees, 2004) and secretion of accessory molecules that strengthen the interaction between lipoproteins and proteoglycans (Gustafsson et al., 2007; Fogelstrand and Boren, 2012). Consequently, the retention process is amplified, which in turn, boosts the cycle of lipoprotein retention, oxidative and enzymatic modifications, blood-born immune cell accumulation, foam cell formation and T cell subendothelial activation and complicated plaque formation (Tabas et al., 2007; Hansson and Hermansson, 2011; Libby et al., 2013; Garrido-Urbani et al., 2014; Tabas et al., 2015; Tabas and Bornfeldt, 2016). Yet, as the mechanisms leading to lipoprotein retention in the intima may differ between early and late phases of the disease (Tabas et al., 2007), it remains to be shown whether treatment with chP3R99 at later stages could be an effective approach to reduce progression and/or elicit atherosclerotic lesions regression. The present study aims to investigate the vasculoprotective effect of chP3R99 administration in an advanced atherosclerosis setting in apoE^-/-^ mice and to characterize the main mechanisms involved.

## Materials and Methods

### Mice and Treatment Protocol

Eight-weeks-old male apoE^-/-^ mice were fed a HFHC diet (D12108, cholate-free AIN-76A semipurified diet, Research Diets Inc., New Brunswick, NJ, United States) during 11 weeks, at which time they were switched to a normal chow diet (Teklad Rodent 18% protein, Indianapolis, IN, United States), to promote a milder hypercholesterolemic scenario, and the administration protocol was initiated. Food and water were provided *ad libitum*. From 19 weeks of age, apoE^-/-^ mice (10 per group) were treated weekly with three *s.c.* injections of PBS or 50 μg of either chP3R99 or isotype-matched control IgG1 antibody (hR3), followed by two additional injections at 24 and 26 weeks of age. Blood samples were drawn under brief isoflurane anesthesia before treatment and 1 week after the first and the fifth mAb injection for antibody reactivity assay in mouse sera. At 28 weeks of age, mice were euthanized with CO_2_ asphyxiation followed by cardiac puncture exsanguination, after which an intracardiac perfusion with PBS was performed to wash the vascular system for morphometric, immunohistochemical (IHC) and molecular biology evaluations. A third group of untreated apoE^-/-^ mice was sacrificed at 19 weeks of age to determine the extent of arterial lesions before starting treatment. All experimental procedures were approved by the Institutional Animal Ethics Committee, in accordance with the Canadian Council on Animal Care guidelines for use of experimental animals, and conforming to the Guide for the Care and Use of Laboratory Animals published by the US National Institutes of Health (A5213-01).

### Morphometric and IHC Analysis

Morphometric analysis was performed on *en face* oil-Red O-stained aortas (aortic arch and thoracic arteries), as described previously (Febbraio et al., 2000). Lesion areas were digitized by videomicroscopy, analyzed using Adobe Photoshop CS4 software and expressed as the percentage of the aortic surface area covered by lesions. For IHC analysis of the brachiocephalic artery (BCA), the artery was embedded in paraffin and cross-sections of 6 μm were obtained using a microtome along the length of the specimen. Immunostaining was performed in deparaffinized cross-sections after blocking endogenous peroxidase activity, by adding antibodies to CD107b (Mac-3) (clone M3/84, BD Biosciences, San Jose, CA, United States) diluted 1:10 and incubating for 1 h at room temperature). After labeling the anti-Mac-3 primary antibody with biotinylated rabbit anti-rat immunoglobulins (E0468, Dako, Troy, MI, United States), diluted 1:200, specific staining was developed using horseradish peroxidase-conjugated streptavidin (streptavidin-HRP) and diaminobenzidine chromogen (LSAB2 System-HRP, Dako) according to manufacturer’s instructions. CD4 immunostaining was revealed using biotinylated antibodies (clone GK 1.5, R&D Systems, Minneapolis, MN, United States), diluted 1:10 and incubated overnight at 4°C. Immunostained sections were counterstained with Mayer’s hematoxylin and images were acquired using the 20X objective on an Olympus BX51 microscope equipped with a DP70 digital camera. The MAC-3^+^ and CD4^+^ staining were quantified using image analysis software (MATLAB R2012a and Amira 5.2.0) and expressed as percentage of positive staining control (defined as the average staining detected in cross-sections from PBS-treated group).

### Reactivity of Sera against Chimeric Antibodies

The detection of serum autologous antibodies against chP3R99 and the isotype-matched control hR3, as evidence of the generation of a immune response against administrated chimeric mAbs, was determined by solid-phase ELISA as previously described (Brito et al., 2012) with slight modifications. Briefly, 96-well Maxisorp polystyrene plates (Nunc, Thermo Fisher Scientific, Mississauga, ON, Canada) were coated with 10 μg/mL of chP3R99 or hR3 and after an overnight incubation at 4°C, non-specific binding sites were blocked with 1% of bovine serum albumin. Next, mice sera collected before (preimmune) and 1 week after the first and the last administration of chP3R99 (diluted 1/400) were added and the plates were incubated for 1 h at 37°C. Peroxidase-conjugated goat anti-mouse IgG polyclonal secondary antibodies (Jackson ImmunoResearch Laboratories, West Grove, PA, United States) were used to determine the reactivity associated with IgG fractions using 3,3′,5,5′-tetramethylbenzidine (TMB Liquid Substrate System for ELISA, Sigma-Aldrich, Oakville, ON, Canada) for colorimetric reaction development. Reactivity of mice sera vs. hR3 was indicative of the induction of murine antibodies against the isotype of chimeric mAbs (anti-isotypic response) whereas recognition of chP3R99 by mice sera accounted for the presence of anti-idiotype (Ab2, anti-idiotypic response) and anti-isotype antibodies generated by chP3R99 administration. A 2.5-fold or higher increase in levels of anti-chP3R99 response with respect to the anti-isotype ones was the threshold to establish the immunodominance of the Ab2 response. Serum Reactivity was expressed as arbitrary units after background correction. Arbitrary units were defined as the ratio of optical density (OD) values obtained for each serum sample to the average of OD values from normal sera samples (*OD Serum sample/Mean OD normal sera*). Assays were performed in triplicate for each sample and the coefficient of variation was <15% for all values.

### Determination of Sera Reactivity to Heparin

The presence of murine autologous antibodies against heparin, used as sulfated GAG prototype to detect the presence of anti-GAG anti-anti-idiotype (Ab3) antibodies in response to chP3R99 administration, was assessed, in mice sera, by a solid-phase ELISA described previously (Brito et al., 2012) with slight modifications. Briefly, 96-well Maxisorp polystyrene plates (Nunc, Thermo Fisher Scientific, Mississauga, ON, Canada) were coated with 10 μg/mL of heparin and incubated overnight at 4°C. After blocking non-specific binding sites with 1% of bovine serum albumin, mice preimmune sera and the ones collected 7 days after the first and the last administration of chP3R99 or hR3 (diluted 1/400) were added to the plates and incubated for 1 h at room temperature. To determine the reactivity associated to serum IgG fraction, peroxidase-conjugated goat anti-mouse IgG polyclonal secondary antibodies (Jackson ImmunoResearch Laboratories, West Grove, PA, United States) were used and colorimetric reaction was developed using 3,3′,5,5′-tetramethylbenzidine (TMB Liquid Substrate System for ELISA, Sigma-Aldrich, Oakville, ON, Canada). Serum Reactivity was expressed as arbitrary units after background correction. Arbitrary units were defined as the ratio of optical density values obtained for each serum sample to the average of OD values from normal sera samples (*OD serum sample/Mean OD normal sera*). Assays were performed in triplicate for each sample and the coefficient of variation was <15% for all values.

### Total Cholesterol Assay

Total cholesterol was measured in mice sera after 12 h of fasting using the Thermo Scientific^TM^ Total Cholesterol Reagents (Thermo Fisher Scientific).

### Interleukin-6 Assay

The concentration of interleukin-6 (IL-6) in sera was assessed by a mouse ELISA kit (Ready-SET-Go, # 88-7064, eBioscience, San Diego, CA, United States) following manufacturer’s instructions.

### Gene Expression of Inducible Nitric Oxide Synthase and Interleukin-10 in Aorta

Total RNA from the abdominal aorta and iliac arteries was isolated using Trizol (Invitrogen, Carlsbad, CA, United States). Following treatment with DNase I (Invitrogen), purified RNA was used as template for first-strand cDNA synthesis using random primers and M-MLV reverse transcriptase (Invitrogen). Quantitative real-time RT-PCR was run on Rotor-Gene 3000 (Corbett Research, Mortlake, NSW, Australia) system using Sso Fast EvaGreen from Bio-Rad (Hercules, CA, United States) and specific primers of genes including inducible nitric oxide synthase (iNOS) (forward primer, TGCCCTGCTTTGTGCGAAGTGT, reverse primer, TTGAGCCCTTTGTGCTGGGAGTCA) and interleukin-10 (IL-10) (forward primer, GCTCTTGCACTACCAAAGCC, reverse primer, CTGCTGATCCTCATGCCAGT) as positive-controls for M1 and M2 markers gene expression, respectively, and beta-actin (forward primer, AGCAGGAGTACGATGAGTCC, reverse primer, CAGCTCAGTAACAGTCCGC) as internal control. Relative gene expression was calculated using the comparative Ct (2^-ΔΔC_t_^) method.

### Localization of Anti-VCAM-1 Coupled to FITC by Optical Imaging

FITC-conjugated rat anti-mouse vascular cell adhesion molecule-1 (VCAM-1) monoclonal antibody was obtained from LifeSpan BioSciences (Seattle, WA, United States). Twenty-eight weeks-old apoE^-/-^ mice from chP3R99- and hR3-treated groups were shaved in the dorsal area and scanned to determine autofluorescence with the optical imager Optix MX3 System (ART, Advanced Research Technologies Inc, Montreal, QC, Canada). Mice were then injected *i.v*. with 10 μg of anti-VCAM-1-FITC through the subclavian vein, and scanned 72 h later. Images were analyzed using OptiView software (version 3) (ART) and mean fluorescence intensity was expressed in normalized counts (NC). NC are defined as a map representation calculated as photon counts (PC)/[laser intensity (μW) × integration time (s)].

### Statistical Analysis

All data are represented as mean ± SEM. Comparison between independent groups were performed using Student’s *t*-test or Mann–Whitney *U* test to non-parametric data, or unpaired one-way ANOVA followed by Tukey’s method for multiple *post hoc* comparisons (GraphPad Prism Version 4.0c, GraphPad. San Diego, CA, United States). Probability values < 0.05 were considered statistically significant.

## Results

### chP3R99 Treatment Arrested Progression of Advanced Atherosclerotic Lesions without Modulating the Total Cholesterol Levels in *apoE*^-/-^ Mice

**FIGURE 1:**
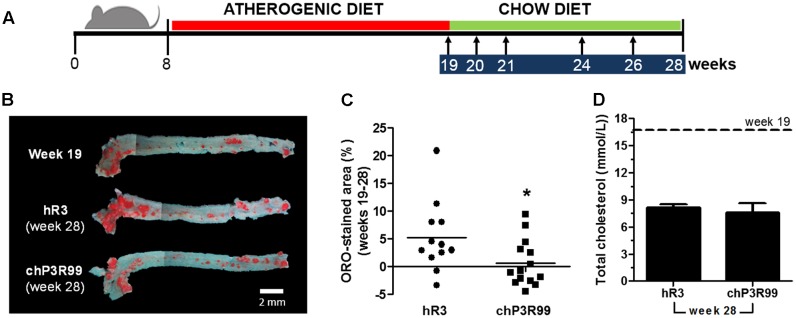
Effect of a 5-dose administration schedule using chP3R99 in *apoE*^-/-^ mice with advanced atherosclerotic lesions. **(A)** Experimental design. Red/Green and blue bars depict two different diet periods and administration schedule, respectively. Arrows indicate administration time points. **(B)** Representative *en face* Oil Red O-stained aortic arches and thoracic aortas from 19-week-old non-treated *apoE*^-/-^ mice (*n* = 11 in total) and 28-week-old *apoE*^-/-^ mice treated with chP3R99 (*n* = 14) or isotype-matched control hR3 (*n* = 12). **(C)** Percent-aortic lesion areas (mean and individual) over baseline (week 19) in mice treated until week 28 with chP3R99 or hR3. **(D)** Total plasma cholesterol in apoE^-/-^ mice at beginning (week 19, dashed line) and end (week 28, bar graphs) of treatment. Results are mean ± SEM of one representative of at least two independent experiments with similar results. ^∗^represents *p* < 0.05 vs. isotype-matched control hR3, unpaired student’s *t*-test.

Atherosclerosis was developed through 11 weeks of HFHC diet (from 8 to 19 weeks of age). Atherosclerotic mice were then switch to regular chow and were injected *s.c*. with hR3 (IgG control) or chP3R99, according to protocol presented in **Figure 1A**. Representative longitudinal sections of mouse aortas (aortic arch and thoracic aorta) stained with oil Red-O (**Figure 1B**), show deposition of lipids mainly to the aortic arch in all groups and similar progression of lesions between week 19 (prior to treatment initiation) and week 28 in hR3- or PBS-treated groups (not shown), despite a return to chow diet. Morphometric quantification of atherosclerotic lesions shows reduced lesion progression by a mean value of 88% (*p* < 0.05) in chP3R99- compared to hR3-treated groups (**Figure 1C**). Analysis of individual mouse lesion areas revealed that treatment with chP3R99 not only abrogated lesion progression, but also initiated regression in a significant number of mice (**Figure 1C**). HFHC-elicited hypercholesterolemia (dotted line) was reduced by 2-fold at week 28 (**Figure 1D**); chP3R99 did not modulate further total cholesterol serum levels at week 28 (**Figure 1D**).

### chP3R99 Administration Elicited Anti-Idiotype (Ab2) and Anti-Sulfated GAGs Antibody Responses

**FIGURE 2:**
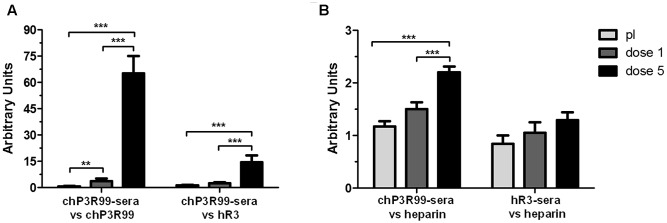
Induction of anti-idiotype and anti-heparin antibodies in apoE^-/-^ mice treated with chP3R99. Sera (diluted 1:400), collected before starting treatment (pI, preimmune) and 7 days after the first and the last administration of chP3R99 or isotype-matched control hR3, were added to chP3R99/hR3- or heparin-coated ELISA plates. Specific antibodies were revealed by peroxidase-conjugated goat anti-mouse IgG polyclonal secondary antibodies. **(A)** Reactivity of sera from chP3R99-treated mice against chP3R99 (anti-idiotype + anti-isotype response) or hR3 (anti-isotype response). A ratio of chP3R99-associated reactivity to hR3-associated reactivity of 2.5-fold or higher was considered as immunodominant induction of anti-idiotype antibodies. **(B)** Reactivity of sera from chP3R99- or hR3-treated mice against heparin. Reactivity levels are expressed as arbitrary units. Results are mean ± SEM. The data are representative of three independent experiments. ^∗∗^*p* < 0.01, ^∗∗∗^*p* < 0.001, one-way ANOVA followed by Tukey’s *post hoc* comparisons.

Low levels of serum IgG antibodies against chP3R99 whole molecule (anti-idiotype and anti-isotype response) were detected 7 days after the first administration with this chimeric mAb (*p* < 0.01, respect to preimmune sera). This response continued to increase until reaching significantly higher levels at the time of the last mAb administration (*p* < 0.001) (**Figure 2A**). Conversely, significant IgG reactivity to hR3 isotype in the same sera from chP3R99-treated mice was observed only in those corresponding to the last mAb administration (*p* < 0.001) (**Figure 2A**). The fact that reactivity to chP3R99 whole molecule was 4.5 times higher than the one sensed against the isotype, suggested the presence of predominant levels of anti-idiotype (Ab2) antibodies in the immune response raised by this chimeric mAb.

On the other hand, the level of murine autologous antibodies against heparin, measured as serum reactivity to this sulfated GAG, was not modulated in hR3-treated mice after the first or last administration of this isotype-matched control (**Figure 2B**). In contrast, baseline reactivity to heparin did not increase in sera from mice treated with one administration of chP3R99, but sera collected after the last mAb administration exhibited increased binding to heparin compared to preimmune sera (*p* < 0.001) (**Figures 2B**).

### chP3R99 Treatment Induced a Striking Reduction of Macrophages and T Cells Accumulation in Brachiocephalic Artery Lesions

**FIGURE 3:**
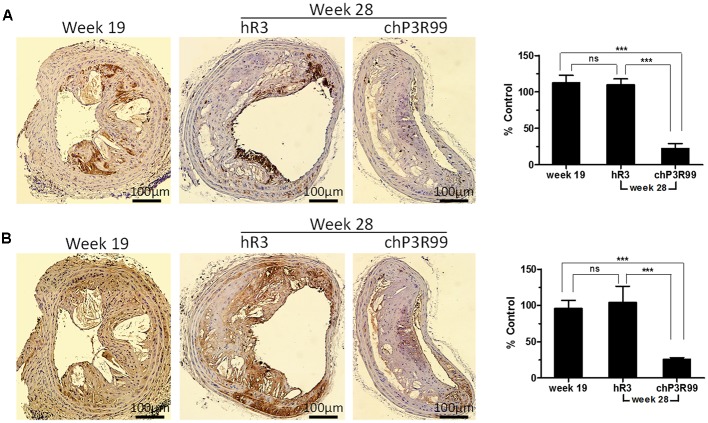
Macrophage and T helper lymphocyte content in advanced plaques of apoE^-/-^ mice after administration of chP3R99. Immunohistochemical staining of representative BCA artery cross-sections from 19-week-old non-treated mice and 28-week-old apoE^-/-^ mice treated with chP3R99 or isotype-matched control hR3 were performed using primary antibodies to cell surface markers. **(A)** CD107b^+^ (Mac-3^+^) macrophage staining and **(B)** CD4^+^ helper T cell staining. Bar graphs show the percentages of CD107b^+^- **(A)** and CD4^+^-staining **(B)** relative to average staining of positive control (PBS-treated group). Data are mean ± SEM of 5 mice per group. A representative result of two independent experiments is shown. ^∗∗∗^*p* < 0.001, one-way ANOVA followed by Tukey’s *post hoc* comparisons.

Brachiocephalic artery cross-sections were stained with either anti-CD107b (Mac-3) or anti-CD4 antibodies to localize sites of macrophage and lymphocyte enrichment, respectively. Representative cross-sections from preimmunized (week 19) and mice treated with IgG control hR3 or chP3R99 (week 28) are shown in **Figures 3A,B**. Quantitative analysis showed that CD107b^+^ staining was similar in BCA cross-sections from mice of 19 weeks of age (preimmunization) and in 28-weeks-old mice which were fed with normal chow diet for nine more weeks and treated with the isotype-matched IgG control (**Figure 3A**). In contrast, administration of chP3R99 markedly decreased CD107b^+^ immunostaining in BCA cross-sections of mice at 28 weeks of age by 80% compared to both 19-weeks-old baseline group and 28-weeks-old hR3-treated group (*p* < 0.001) (**Figure 3A**). In a similar manner, CD4^+^ lymphocytes staining were elevated in both 19- and 28- (hR3-injected) weeks-old mice, while being strikingly reduced in chP3R99-treated mice, by 75% compared to baseline and hR3-treated mice at 28 weeks of age (*p* < 0.001) (**Figure 3B**).

### chP3R99 Administration Reduced Pro-Inflammatory IL-6 Cytokine Levels in Sera and Increased the Expression Ratio of IL-10/iNOS in Abdominal Aortas and Iliac Arteries

**FIGURE 4:**
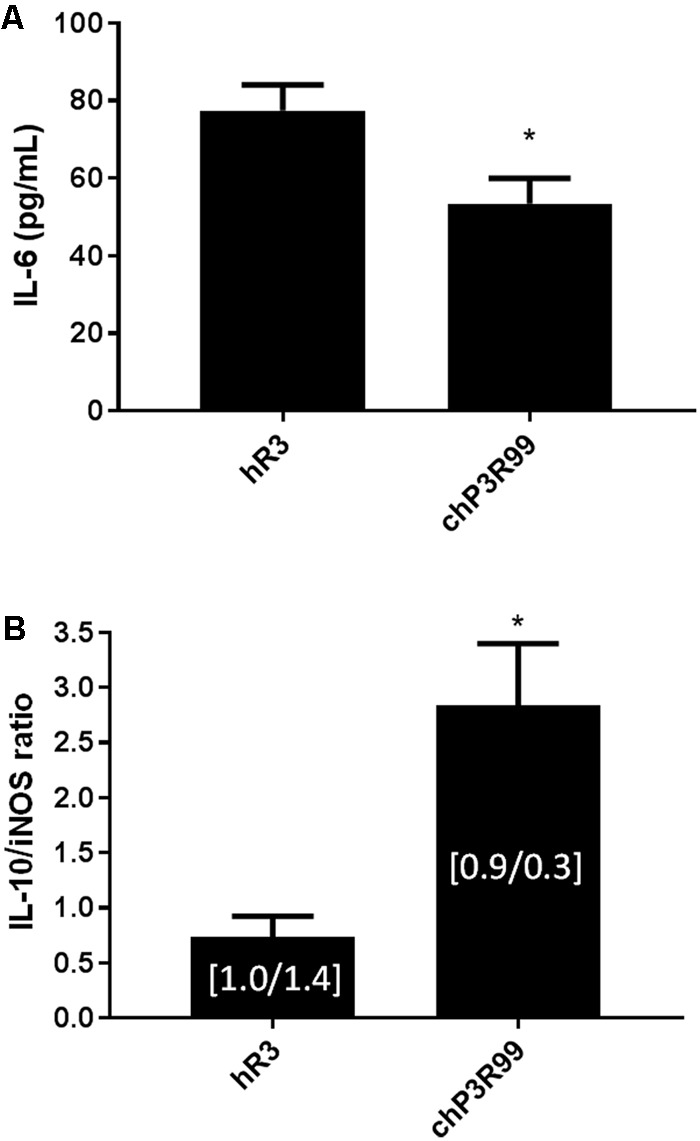
Effect of chP3R99 administration on circulating IL-6 levels and on the ratio of aortic IL-10 to iNOS expression. **(A)** IL-6 concentrations in sera at week 28 in mice treated with chP3R99 or isotype-matched control hR3. **(B)** Bar graphs show the ratio of aortic IL-10 to iNOS mRNA levels in chP3R99 or isotype-matched control hR3 as assessed by qPCR. Data are mean ± SEM of 10 (IL-6) and 5 (IL-10/iNOS ratio) mice per group. Inserts in bar graphs are the mean values of relative IL-10 and iNOS gene expression, respectively. Quantitative data are representative of two independent experiments. ^∗^represents *p* < 0.05 vs. isotype-matched control hR3, Mann–Whitney *U* test.

IL-6 concentrations in sera were diminished by 31% (*p* < 0.05) in chP3R99-treated *apoE*^-/-^ mice compared to hR3-treated animals at 28 weeks of age (**Figure 4A**). The average ratio of IL-10 to iNOS expression levels in aortic homogenates of abdominal aortas and iliac arteries was significantly elevated by more than 3-fold in 28-week-old chP3R99-treated mice as shown in **Figure 4B**.

### chP3R99 Treatment Reduced VCAM-1-FITC Staining *in Vivo*

**FIGURE 5:**
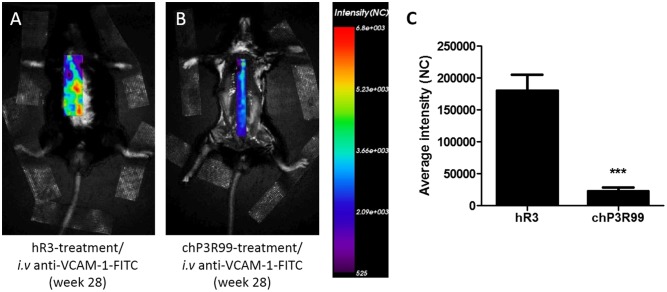
*In vivo* localization of anti-VCAM-1 mAb coupled to FITC in 28-week-old apoE^-/-^ mice. At the end of the administration protocol, mice received an *i.v.* injection of anti-VCAM-1-FITC mAb (10 μg) and were scanned before (for autofluorescence background detection) and 3 days after anti-VCAM-1-FITC administration by optical imager Optix MX3 System. **(A)** Representative scan of a mouse from hR3-treated group. **(B)** Representative scan of a mouse from chP3R99-treated group. **(C)** Bar graph representing mean intensity expressed in normalized counts (NC) ± SEM (*n* = 4 mice per group). ^∗∗^*^∗^*represents *p* < 0.001 vs. isotype-matched hR3, unpaired student’s *t*-test.

Twenty-eight weeks-old *apoE*^-/-^ mice treated with chP3R99 or control hR3 were injected *i.v.* with 10 μg of anti-VCAM-FITC and scanned with an optical imaging system 72 h later. Representative scans of mice are shown in **Figure 5A** (hR3-treated mice) and **Figure 5B** (chP3R99-treated mice) along with the fluorescence intensity scale bar. The bar graph in **Figure 5C** represents the average intensity in the two experimental groups, showing a striking reduction of anti-VCAM-1-FITC fluorescence in chP3R99-treated mice.

## Discussion

Most preclinical studies to date have the limitation, with few exceptions, to have shown benefits of potential therapeutic candidates in the early phase of atherosclerosis development rather than in advanced disease, although the later may be more relevant to human disease (Bjorkbacka et al., 2013). In the present study, we show that treatment with chP3R99, a chimeric mouse/human monoclonal antibody recognizing sulfated GAGs, halts lesion progression and triggers lesion remodeling and regression in advanced atherosclerotic lesions. Our previous studies on chP3R99 administration in a model of atherosclerotic plaque progression, in which *apoE*^-/-^ mice were maintained on a HFHC diet throughout the study, showed the preventive (Brito et al., 2012) or curative effect of the treatment in reducing the progression of less advanced atherosclerotic lesions (Delgado-Roche et al., 2015). These vasculoprotective effects were likely associated with the induction of anti-anti-idiotypic antibodies against sulfated GAGs, able to block the interaction of intimal LDL with proteoglycans. Yet, it remained unclear whether administration of chP3R99 would be atheroprotective at advanced stages of disease and may elicit lesion regression.

To this purpose, *apoE*^-/-^ mice were fed a HFHC diet until 19 weeks of age to accelerate lesions development to an advanced stage (Nakashima et al., 1994; Getz and Reardon, 2012) and then resumed a normal chow diet while administered five doses of chP3R99 between 19 and 28-weeks of age. At this phase of development, plaques have highly retentive extracellular matrix owing to secretion of GAGs with higher affinity for apoB (Chang et al., 2000). Furthermore, production of accessory molecules like sphingomyelinase, which promotes LDL aggregation (Devlin et al., 2008) and lipoprotein lipase that serves as a bridge between LDL and GAGs (Gustafsson et al., 2007), further enhance the retention process. Our results show that after switching to a chow diet, plasma total cholesterol dropped by 50%; however, mice remained hypercholesterolemic and in consequence, lesions continued to increase in size after week 19 in PBS- and isotype-matched control hR3-groups, particularly at the aortic cross-level (**Figure 1B**). This allowed us to evaluate the effect of the treatment with chP3R99 to stop the overall lesion progression in advanced disease under an environment of mild hypercholesterolemia. Interestingly, administration of chP3R99 promoted a reduction in lesion area below baseline at week 19 in a large number of mice (8 of 14-chP3R99 vs. 2 of 11-hR3), suggesting that the treatment may impact plaque progression and regression pathways in these mice.

The present results differ from data obtained in our previous study of chP3R99-therapeutic treatment (Delgado-Roche et al., 2015). In the former work, in which atherogenic diet was maintained throughout the protocol (progression scenario), treatment was started in the early to intermediate phases of atherosclerotic lesions development (week 10) and extended throughout 8 weeks. In this condition, chP3R99 low-dose administration halted the progression of established disease, maintaining the overall aortic lesion area reached at week 10, stable over treatment period. Nevertheless, overall disease did not decrease at levels below the baseline in any of the treated mice, indicating failure to promote regression of early/intermediate atherosclerosis in this environment of severe hypercholesterolemia (Delgado-Roche et al., 2015). In this new study, first, results are achieved in a more advanced atherosclerosis scenario with an overall aortic lesion area at treatment baseline 2-fold higher compared to former work. Second, current data support therapeutic potential of the administration of chP3R99 mAb to impact both plaque progression and regression pathways in late atherosclerosis under conditions of mild hypercholesterolemia. Third, our results provide new evidence validating GAGs as a target for the impairment of lipoprotein retention in late atherosclerosis, in opposition to previous finding indicating that the atheroprotective capacity conferred by mutations of LDL that reduce its proteoglycan-binding capacity (which can resemble the blocking of LDL binding site in GAGs) is abolished at advanced stages of disease (Gustafsson et al., 2007).

In agreement with our previous reports (Brito et al., 2012; Soto et al., 2012; Delgado-Roche et al., 2015) the immunological evidences obtained in this work suggest, in this advanced scenario, the generation of anti-GAG anti-anti-idiotype (Ab3) antibodies rendered by an anti-idiotype cascade established through chP3R99 administration. First, analysis of the differences in the reactivity of sera from chP3R99-treated mice against this mAb and its isotype control, pointed out to the induction of an immunodominant response against chP3R99 idiotype (Ab2 antibodies) increasing with the number of doses administered. Second, detection of anti-heparin antibody levels in sera from chP3R99-treated mice and not in those from mice that received hR3 suggested that induced anti-GAG response, probably Ab3, is linked to chP3R99 idiotype and the Ab2 antibodies generated by its administration, as was previously demonstrated (Brito et al., 2012). Third, delay in the induction of anti-heparin response with respect to Ab2 response suggested, once again, that both processes occurred sequentially, supporting the anti-idiotype cascade hypothesis. Of note, anti-heparin levels detected in serum, in this scenario, were lower than those observed in a previous study (Brito et al., 2012), result that might be related with a higher accumulation of induced antibodies in aortic lesions with the consequent establishment of a tissue-blood equilibrium in which circulating antibodies are diminished. Additionally, reactivity levels to heparin (in terms of OD values) in preimmune sera as well as in normal sera from age-matched apoE^-/-^ mice were higher than those observed in previous studies, which is consistent with literature reports indicating an increase in serum IgG1 levels in advanced atherosclerosis due to a greater activation state of B lymphocytes (Rincón-Arévalo et al., 2016). As we stated in previous studies (Brito et al., 2012; Delgado-Roche et al., 2015), the antiatherosclerotic effect exhibited by chP3R99 administration in this advanced scenario was likely related, in a large degree, to the generated anti-GAG response, first, because contribution of a protective passive effect should have been minimal at administered dose (50 μg). Second, ELISA performed to detect anti-human antibodies failed to detect chP3R99 in mice sera (not shown), suggesting high clearance rates of this mAb from circulation. Consequently, it is unlikely that stable therapeutic levels of chP3R99 have been achieved during treatment. Nevertheless, a passive contribution of chP3R99 to the atheroprotective effect cannot be neglected, particularly during the first three weekly administrations. Future studies must be conducted to conclusively demonstrate the correlation between the induced anti-GAG response and the antiatherosclerotic effect. In addition, assessment of total plasma cholesterol confirmed no intervention in lipid metabolism.

Although few preclinical studies have documented stabilization and regression of pre-established/advanced atherosclerotic lesions in conditions of mild or severe hypercholesterolemia, most of them have been based on passive antibody strategies or pharmacological treatment with high doses and high frequencies of treatment (Nakajima et al., 2011; Hayashi et al., 2012; Herbin et al., 2012; Kita et al., 2014; Gabunia et al., 2016; Zimmer et al., 2016). To our knowledge, this study is the first to demonstrate reduced progression of advanced atherosclerotic lesions in hypercholesterolemic *apoE*^-/-^ mice by means of a therapy against GAGs. In addition, evidence provided in this work point out that induction of anti-GAGs antibodies that block lipoprotein retention could inhibit the progression of disease and promote its regression in a stage in which the vicious cycle of enhanced LDL retention-modification and inflammation is amplified.

Since atherosclerosis entails a massive failure in resolution of inflammation triggered by subendothelial retention of apoB-containing lipoproteins (Libby et al., 2014; Tabas et al., 2015), successful antiatherosclerotic therapies must influence, either directly or indirectly, subendothelial inflammation. Along this line, we examined the effect of chP3R99 treatment on macrophage and T-helper lymphocyte infiltration in advanced lesions of BCA, a predilection location for atherosclerosis development (Nakashima et al., 1994). After 11 weeks of atherogenic diet, at 19 weeks of age, infiltration of CD4^+^ T-lymphocytes and macrophages, were observed in lesional intima and localized zones of media layer. Accumulation of immune cells remained the same at 28 weeks of age in mice treated with the IgG control hR3. This could be due to the effect of decreased hypercholesterolemia in spite of a more advanced state of lesions in BCA.

Interestingly, administration of five doses of chP3R99 dramatically reduced infiltration of both macrophages and T-helper cells between 75 and 80% compared to baseline (week 19) and hR3 controls. Many studies focused on atherosclerosis regression have revealed that reduction in lesion size is accompanied by a drastic decrease in plaque macrophage content (Reis et al., 2001; Trogan et al., 2004; Raffai et al., 2005; Shaw et al., 2008). The proposed mechanisms include CCR7-dependent emigration of macrophages to draining and systemic lymph nodes (Llodra et al., 2004; Trogan et al., 2006; Feig, 2014) and macrophage apoptosis and efferocytosis, together with cessation in monocyte recruitment (Potteaux et al., 2011; Randolph, 2014). Alternatively, administration of anti-CD3 antibody was shown to induce regression of established plaques via reducing CD4^+^ T cells number along with an increase in the proportion of regulatory T (Treg) cells (Kita et al., 2014). Similar findings have been obtained in *apoE*^-/-^ mice infused with apoB 100-derived peptides, for which a decrease in plaque progression was associated with reduction of T cell infiltration and to an induction of a Treg cell response (Herbin et al., 2012). In line with these observations, our finding that chP3R99 drastically reduced T cell and macrophage content in advanced lesions may support the hypothesis of a regression mechanism linked to its antiinflammatory properties. The process of regression, following the abrogation of inflammatory cell infiltration, has been reported to require a stream of healthy phagocytes that remove necrotic debris and other components of the plaques (Feig, 2014), setting a time frame between inflammation resolution and regression of lesion size, which seems to be larger in more advanced stages (Feig et al., 2008; Williams et al., 2008). Considering that BCA is one of the first sites to develop lesions that progress to advanced stage (Nakashima et al., 1994), this phenomenon may explain apparent similarity in size among BCA lesions of hR3- and chP3R99-treated mice at week 28. Thus, it is likely that even though at the end of treatment period supporting evidences of a regression process were observed, this time frame was not larger enough to verify a regression in size of advanced BCA lesions. Future studies must be conducted to overcome this limitation and to further dissect the mechanisms involved in chP3R99-elicited regression.

The balance between antiinflammatory and proinflammatory cytokines is crucial for lesion fate, where the shift of equilibrium toward the latter is usually associated to atherosclerosis development (Kleemann et al., 2008). In fact, potential therapies for the treatment of atherosclerosis such as mucosal administration of athero-antigens (HSP 65, oxLDL, apoB-100 peptides) have shown increments in production of IL-10, IL-4 and TGF-β (Harats et al., 2002; van Puijvelde et al., 2006; Klingenberg et al., 2010). Likewise, active immunization against specific microbes of gut microbiota has demonstrated to promote an apoE-mediated reduction of circulating levels of proinflammatory cytokines and the impairment of atherosclerosis and western diet-related inflammatory state in general (Saita et al., 2016). Moreover, macrophages within lesions feature impressive plasticity, adopting either an inflammatory (M1) phenotype with an increase in pro-inflammatory cytokine and chemokine production and of inducible nitric oxide synthase (iNOS), or a proresolving (M2) phenotype, exemplified by IL-10 production (Tabas and Bornfeldt, 2016). Both M1 and M2 phenotypes coexist in lesions, and an imbalance between the M1 and M2 phenotype may explain impaired resolution (Tabas, 2010). For instance, the resistance to atherosclerosis observed in *Irgm1*-haplodeficient mice was associated to a decrease in M1-macrophage content in lesions along with reduction in both iNOS expression and M1 polarization-related transcription factors, with negligible impact on the M2 phenotype (Fang et al., 2016). In our study, treatment with chP3R99 increased the ratio of IL-10/iNOS, along with reduced circulating levels of IL-6. These observations support reduced preponderance of inflammatory genes as a direct consequence of a massive decrease in subendothelial inflammatory cell number as observed in advanced BCA lesions. Consistent with these findings, 28-week-old chP3R99-treated mice exhibited a marked reduction in the fluorescence signal detected after the *i.v.* injection of an anti-VCAM-1 antibody coupled to FITC compared to hR3-treated mice, indicating an attenuation of the inflammatory active state of endothelium in lesions, which coincide with the effect of treatment on reduced mononuclear leukocyte recruitment. Indeed, VCAM-1 is an adhesion molecule that is only expressed on inflamed endothelium (Ley and Huo, 2001). This molecule plays a critical role in atherosclerosis by mediating extravasation of monocytes and T cells (Andersson et al., 2010). The reduction of anti-VCAM-1 labeling in mice treated with chP3R99 mAb suggests the attenuation of the inflammatory active state of endothelium in atherosclerotic lesions. This effect was likely promoted by a decrease in both oxLDL accumulation and inflammatory cytokine secretion, stimuli that upregulate VCAM-1 expression on endothelial cells during atherosclerosis onset and progression (Andersson et al., 2010).

## Conclusion

Our research provides evidence that therapeutic administration of chP3R99, an anti-GAG antibody, stops lesion progression and orchestrates processes that precede and may lead to regression of advanced atherosclerotic lesions as a result of a drastic lessening of subendothelial inflammation. This effect remains probably related to generation of autologous anti-GAG antibodies that block lipoprotein retention, therefore pointing to the lipoprotein retention process as a key target for therapeutic intervention at later stages of atherosclerosis.

## Author Contributions

VB, KM, KZ, LM, and RS performed the experiments and/or assays. YS performed data analysis and critical reading of the manuscript. VB, HO, SM and AV designed the study, contributed to data analysis and wrote the paper. LF performed critical reading of the manuscript.

## Conflict of Interest Statement

AV, VB, and YS are inventors of patents related with P3 monoclonal antibody and its anti-idiotype and to antibodies that recognize sulfatides and sulfated proteoglycans; however, the inventors have signed the assignment of their rights to the assignee Center of Molecular Immunology. The other authors declare that the research was conducted in the absence of any commercial or financial relationships that could be construed as a potential conflict of interest.
